# Mechanisms and roles of mitophagy in neurodegenerative diseases

**DOI:** 10.1111/cns.13140

**Published:** 2019-05-02

**Authors:** Yan Wang, Na Liu, Bingwei Lu

**Affiliations:** ^1^ Department of Pharmacology, College of Pharmaceutical Science Soochow University Suzhou China; ^2^ Department of Pathology Stanford University School of Medicine Stanford California

**Keywords:** LC3 adapters, mitochondria, mitophagy, mitophagy receptors, neurodegenerative diseases, Parkin, PINK1, ubiquitin

## Abstract

Mitochondria are double‐membrane‐encircled organelles existing in most eukaryotic cells and playing important roles in energy production, metabolism, Ca^2+^ buffering, and cell signaling. Mitophagy is the selective degradation of mitochondria by autophagy. Mitophagy can effectively remove damaged or stressed mitochondria, which is essential for cellular health. Thanks to the implementation of genetics, cell biology, and proteomics approaches, we are beginning to understand the mechanisms of mitophagy, including the roles of ubiquitin‐dependent and receptor‐dependent signals on damaged mitochondria in triggering mitophagy. Mitochondrial dysfunction and defective mitophagy have been broadly associated with neurodegenerative diseases. This review is aimed at summarizing the mechanisms of mitophagy in higher organisms and the roles of mitophagy in the pathogenesis of neurodegenerative diseases. Although many studies have been devoted to elucidating the mitophagy process, a deeper understanding of the mechanisms leading to mitophagy defects in neurodegenerative diseases is required for the development of new therapeutic interventions, taking into account the multifactorial nature of diseases and the phenotypic heterogeneity of patients.

## 1 INTRODUCTION

As key organelles widely distributed in most eukaryotic cells, mitochondria are indispensable for diverse cellular processes, from generating adenosine triphosphate (ATP), maintaining calcium homeostasis, synthesizing key metabolites, producing endogenous reactive oxygen species (ROS), to regulating necrosis, apoptosis, and autophagy.^1^, ^2^, ^3^ Therefore, appropriate regulation of mitochondrial health is of crucial importance to various physiological and pathological processes. In yeast, deletion of the *Mdm38* gene product, an inner mitochondrial membrane (IMM) protein with K^+^/H^+^ exchanger activity, can reduce the content of respiratory chain complex, induce mitochondrial morphological changes, break the homeostasis of mitochondrial K^+^, and lead to mitophagy.^4^ Mitochondria are removed by the process of autophagy when mammalian reticulocytes mature into erythrocytes.^5^ In fertilized *Caenorhabditis elegans* oocytes, the sperm‐derived mitochondria would be removed by mitophagy.^6^, ^7^ These results suggest that maintaining mitochondrial homeostasis by mitophagy plays an important role in the differentiation and development of eukaryotes.

Mitochondrial dysfunction has been implicated in numerous neurodegenerative diseases including Parkinson's disease (PD). PD is characterized by the degeneration of dopaminergic neurons in the midbrain. Mitochondrial dysregulation can lead to PD.^8^, ^9^, ^10^, ^11^ Mutations in *PINK1* and *Parkin* genes are associated with autosomal recessive early‐onset PD.^12^, ^13^ *PINK1* and *Parkin* genes exert synergistic effect on mitochondrial maintenance‐related functions, such as mitochondrial motility, proteasomal degradation of mitochondrial proteins, and mitophagy. Additionally, Parkin also participates in removing mitochondria during the progression of Alzheimer's disease (AD), and the overexpression of Parkin can alleviate symptoms of AD.^14^ Supporting a role of mitophagy in AD, amyloid beta‐derived diffusible ligands (ADDLs) can induce the fragmentation of mitochondria and mitophagy.^15^, ^16^, ^17^ Mitophagy is also altered in HD, and the mutant huntingtin may induce selective autophagy.^18^ In a cell culture model of HD, excessive mitochondrial fission partially mediates cytotoxicity.^19^ Mitochondrial dysfunction has also been observed in ALS, and reduced targeting of ubiquitinated mitochondria to autophagosomes may contribute to the pathogenesis of ALS.^20^ Mitophagy thus plays a multifaced role in neurodegenerative diseases.

## 2 SELECTIVE AND NONSELECTIVE AUTOPHAGY

Under certain conditions, organelles, together with bits of cytoplasm, will be sequestered and degraded by lysosomes for hydrolytic digestion in a process termed autophagy.^21^ Generally, autophagy can be classified into nonselective autophagy and selective autophagy, with the former being primarily a starvation response, while the latter eliminating damaged organelles and remodeling cells to adapt to environmental changes.^22^ Defects in selective autophagy can result in a range of human pathophysiologies, including certain types of neurodegenerative diseases. Macroautophagy is an evolutionarily conserved process that allows cells to degrade and recycle cytoplasm. Whereas nonselective macroautophagy can randomly engulf a portion of cytoplasm into autophagosome and subsequently transfer it to the vacuole or lysosome for degradation, selective macroautophagy can specifically identify and degrade specific substances, such as protein complexes, organelles, or invading microorganisms.^23^ The morphological hallmark of macroautophagy is the formation of an initial sequestering compartment, the phagophore, which can expand into the double‐membrane autophagosome, and the initial sequestration occurs in a compartment separate from the degraded organelle. Typically, selective macroautophagy can be classified into mitophagy, pexophagy, reticulophagy, ribophagy, etc. In higher eukaryotes, selective autophagy also includes chaperone‐mediated autophagy (CMA), as well as two similar processes, namely endosomal microautophagy (e‐MI) and chaperone‐assisted selective autophagy (CASA).^24^ Nonetheless, how a substrate is targeted for sequestration and segregated from other cellular parts remains one of the major issues in this research field.

## 3 MITOPHAGY: SELECTIVE AUTOPHAGY OF MITOCHONDRIA

The by‐products of mitochondrial metabolism can induce DNA damage or genetic mutation.^25^ Therefore, mitochondrial health is found to play a vital role in cellular health. Mitophagy, a selective autophagy process, is essential for mitochondrial health.^26^ ATP is the product of oxidative phosphorylation, which can result in ROS production within mitochondria. Excessive mitochondrial ROS can cause cytotoxicity and lead to cell death under some conditions. Mitochondria are particularly vulnerable to ROS damage due to the “naked” nature of their DNA and limited antioxidant capacity inside mitochondria. If not properly repaired or cleared from cells, the unhealthy mitochondria will cause further production of ROS and release of proapoptotic proteins into the cytosol, finally leading to a high risk of cell death.^27^

Mitophagy, first discovered by Lewis and Lewis^28^ in cells, is a kind of selective autophagy that handles dysfunctional or damaged mitochondria, which tend to accumulate following mitochondrial damage or stress. Ashford and Porter^29^ employed electron microscopes to observe the mitochondrial debris in 1962, and it was suggested that functional alterations of mitochondria would trigger their autophagy.^30^ The term “mitophagy” came into use in 1998.^31^

Mitophagy is required to remove the unhealthy mitochondria timely and to maintain steady mitochondrial turnover, as well as cellular development and differentiation.^32^ Notably, selective degradation of the surplus or dysfunctional mitochondria by autophagy has been observed in organisms ranging from yeast to mammals. In yeast, mitophagy is mediated by Atg32, which is related to NIX and its regulator Bcl2/adenovirus E1B 19‐kDa protein‐interacting protein 3 (BNIP3) in mammals. Numerous studies in metazoans suggest that mitophagy is mostly regulated by PINK1 and Parkin, which are not present in yeast, suggesting species‐specific difference in mitophagy regulation. Mitophagy is one of the most extensively investigated types of “organelle autophagy,” which can be partially ascribed to the connection between mitophagy and disease.

## 4 MITOPHAGY IN MAMMALS

**Figure 1:**
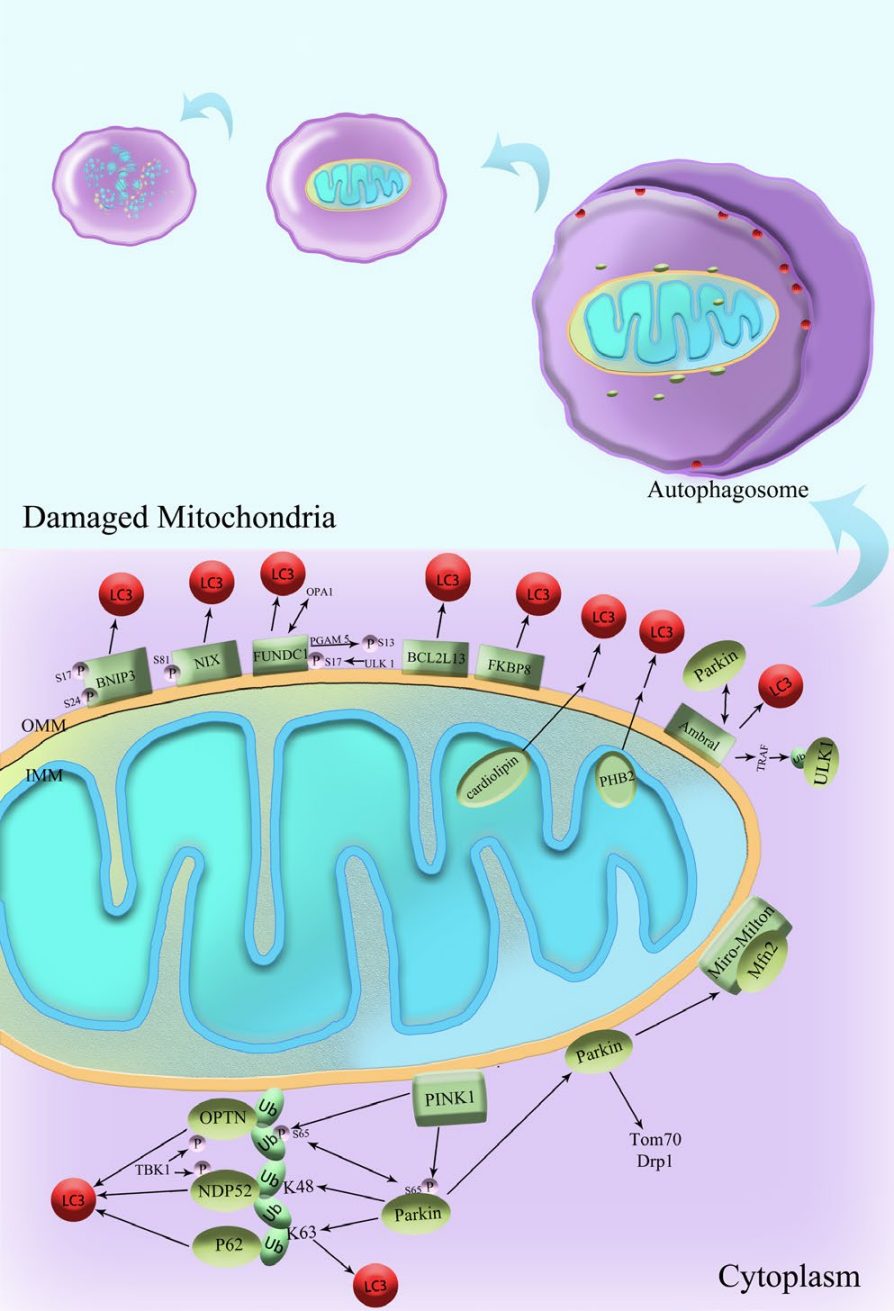
Mechanisms of Mitophagy in mammals. Some OMM proteins, such as BNIP3, NIX, and FUNDC1, possess the LC3‐interacting regions (LIRs) and can interact with LC3. Thus, the interaction between the LIRs of mitophagy receptors and LC3 is considered a crucial link in selecting mitochondria as the cargo. Moreover, reversible protein phosphorylation is suggested to effectively regulate the receptor‐mediated mitophagy. PINK1 and Parkin synergistically regulate mitophagy in mammals in the same pathway. In the damaged mitochondria, mitochondrial membrane potential is lost and PINK1 accumulates on the membrane. PINK1 would recruit Parkin to the damaged mitochondria and phosphorylate Parkin. Parkin can stimulate the ubiquitination of its substrates on OMM via K48 or K63 linkage, followed by protein quality control and subsequent mitochondrial quality control. Many autophagy adaptor proteins are involved in this process, such as p62, NBR1, OPTN, NDP52, and TAX1BP1. Ambra1, activating molecule in beclin 1‐regulated autophagy; BCL2L13, BCL2‐like 13; BNIP3, Bcl2/adenovirus E1B 19‐kDa protein‐interacting protein 3; Drp1, dynamin‐related protein 1; FKBP8, FK506‐binding protein 8; FUNDC1, FUN14 domain‐containing protein 1; LC3, microtubule‐associated protein 1 light chain 3; Mfn 2, mitofusin 2; NDP52 (CALCOCO2), calcium‐binding and coiled‐coil domain 2; NIX (BNIP3L), BCL2/adenovirus E1B 19‐kDa interacting protein 3‐like; OPTN, optineurin; P, phosphate; p62 (SQSTM1), sequestosome 1; PGAM5, phosphoglycerate mutase family member 5; PHB2, Prohibitin 2; PINK1, PTEN‐induced putative kinase 1; TOM70, translocase of outer mitochondrial membrane 70; TRAF, tumor necrosis factor receptor‐associated factor; Ub, ubiquitin; ULK1, UNC51‐like kinase 1

In mammalian cells, mitophagy is activated by two distinct pathways, one is dependent on receptors while the other one relies on ubiquitin (Figure 1). Although both types of mitophagy, namely the receptor‐mediated and PINK1/Parkin‐mediated mitophagy, have been well studied recently, it remains unknown about how these two types of mitophagy differ in expression among different tissues or in output of mitochondrial degradation.^33^ In mammals, both NIX (also known as BNIP3L) and SQSTM1/p62 have been implicated to function as the receptors linking mitochondria with the autophagy mechanism in different cell types. During erythrocyte maturation, NIX is essential for mitochondrial clearance, where mitophagy plays a developmental role. Autophagosome can recognize target mitochondria through LC3 adapters (in ubiquitin‐dependent and ubiquitin‐independent manners) and the direct interaction of LC3 with its receptors.^34^

### 4.1 Mitophagy receptors in mammals

The molecular mechanisms of mitophagy in mammals appear to be quite distinct from that in yeast. The mammalian homolog of yeast Atg32 has not been identified. Some functional counterparts of Atg32, such as BNIP3, NIX, and FUN14 domain‐containing protein 1 (FUNDC1), have been suggested to serve as the mitophagy receptors in mammals.^35^ All these receptors can be recruited to the OMM, and they possess the LC3‐interacting regions (LIRs) and can interact with LC3, a mammalian homolog of Atg8. Thus, the interaction between the LIRs of mitophagy receptors and LC3 is recognized as a crucial link in selecting mitochondria as the cargo. Moreover, reversible protein phosphorylation is suggested to effectively regulate the receptor‐mediated mitophagy. For example, under normoxic conditions, Src and CK2 can phosphorylate FUNDC1 and suppress mitophagy; however, under hypoxia conditions, phosphoglycerate mutase 5 (PGAM5) can dephosphorylate FUNDC1 and thereby effectively promote mitophagy.^36^, ^37^

#### 4.1.1 BNIP3 and NIX/BNIP3L in mammalian mitophagy

BNIP3 and its homologous protein BNIP3L/NIX are BH3‐only proteins, belonging to the BCL2 family. BNIP3 and NIX function in cell death and work together with the LC3 family proteins to remove mitochondria,^38^ with NIX being implicated in reticulocyte maturation.^39^ Atg7 and ULK1 are also involved in mitochondrial removal in reticulocytes. Moreover, nonautophagic mechanisms may also promote mitochondrial removal during reticulocyte maturation.^40^ NIX/BNIP3L and BNIP3, which are regulated by hypoxia‐inducible factor (HIF) or forkhead homeobox type O (FOXO), also participate in the hypoxia‐induced mitophagy.^41^

While the upregulation of NIX or BNIP3 can enhance their activities in mitophagy, the interplay between BNIP3 and LC3 may also act at the level of phosphorylation of BNIP3. When Ser17 and Ser24 adjacent to the LIRs of BNIP3 are phosphorylated, the interplay between BNIP3 and LC3 is enhanced, suggesting a possible role of kinases or phosphatases in regulating mitophagy. Additionally, the interaction between NIX and a small GTPase Rheb is shown to initiate mitophagy.^42^

#### 4.1.2 FUNDC1‐mediated mitophagy

FUNDC1 is an OMM protein associated with the hypoxia‐induced mitophagy. Under hypoxia condition, the FUNDC1 mRNA and protein levels are dramatically downregulated, which may be attributable to mitophagy. No conserved HIF‐1 recognition site is found in the promoter region of FUNDC1.^43^ FUNDC1 can effectively activate the autophagy mechanism and promote mitophagy. The FUNDC1‐dependent mitophagy may also contain a Beclin1‐independent component.^44^ Mutations in the Y(18) and L(21) conserved sites of the FUNDC1 LIRs, together with the phosphorylation of Tyr18 and Ser13 can effectively inhibit the interplay with LC3.^45^

FUNDC1 is phosphorylated at Ser17 by ULK1 and dephosphorylated at Ser13 by PGAM5 under hypoxia condition. BCL2L1/Bcl‐X_L _can not only inhibit the PGAM5 phosphatase activity, but also interact with PGAM5 and block the dephosphorylation of FUNDC1.^46^ Under hypoxia condition, ULK1‐mediated phosphorylation at Ser17 can promote the interaction between FUNDC1 and LC3.^47^ Additionally, the mitochondrial E3 ligase MARCH5 can regulate hypoxia‐induced mitophagy through ubiquitinating and degrading FUNDC1.^48^ The receptor‐interacting serine/threonine‐protein kinase 3 (Ripk3) can suppress FUNDC1‐mediated mitophagy and promote mitochondrial apoptosis in cardiac ischemia/reperfusion injury.^49^ Unexpectedly, it is also suggested that knockdown or overexpression of FUNDC1 has insignificant influence on starvation‐ or hypoxia‐induced mitophagy. The cause of such divergent results remains to be fully clarified.

In mammalian cells, FUNDC1 can recruit LC3 through its LIR motif, thereby activating mitophagy; it can also interact with DNM1L/DRP1 and OPA1, regulating mitochondrial fission or fusion, and mediating mitophagy. OPA1 can interact with FUNDC1 through its Lys70 (K70) residue, and mutation of K70 to Ala (A) will eliminate the interaction between OPA1 and FUNDC1, and promote mitochondrial fission and mitophagy.^50^ Therefore, FUNDC1 can coordinate the dynamics and quality control of mitochondria.

#### 4.1.3 BCL2L13 and FKBP8

Bcl2‐like 13 (BCL2L13), which is located on the OMM and can bind to LC3 via the LIR motif, contains one transmembrane region and one LIR on its N‐terminal facing the cytoplasm. BCL2L13 has been identified as one of the functional counterparts of Atg32, since exogenous BCL2L13 expression can partially rescue a mitophagy defect in the atg32Δ yeast. Similar to the case of Atg32, the phosphorylation of Ser272 on BCL2L13 can also stimulate the binding of BCL2L13 to LC3.^51^, ^52^

FK506‐binding protein 8 (FKBP8) is an LC3‐interacting protein located on the OMM, which can effectively promote mitophagy in a Parkin‐independent manner.^53^ In particular, FKBP8 migrates from mitochondria into ER following treatment with CCCP, a chemical ionophore. The FKBP8 N412K mutant that cannot translocate to ER is defective in suppressing apoptosis during mitophagy, suggesting that not all mitochondrial proteins are degraded during mitophagy, and that the subcellular localization of FKBP8 can regulate cell survival during mitophagy.^54^

#### 4.1.4 PHB2 and cardiolipin

Although prohibitin 2 (PHB2) is located in the IMM, Parkin‐mediated degradation of OMM proteins can trigger the rupture of OMM, exposing PHB2 to LC3 and finally inducing mitophagy. In *C elegans,* PHB2 plays a vital role in the removal of damaged mitochondria.^55^, ^56^

Cardiolipin is a kind of membrane lipid existing in the IMM, which can also serve as a receptor of LC3 in mitophagy. Cardiolipin can be transferred from IMM to OMM during mitochondrial depolarization to induce mitophagy. Cardiolipin and Parkin can independently respond to CCCP treatment and regulate mitophagy at different levels of mitochondrial depolarization.^57^, ^58^

Externalized cardiolipin can directly interact with the N‐terminal helix of LC3, which is specific to the LC3 subtype. In rotenone‐treated cells, cardiolipin can directly interact with gamma‐aminobutyric acid receptor‐associated protein (GABARAP), one of the LC3 family members, but it would not recruit GABARAP to mitochondria. Thus, cardiolipin can interact with GABARAP during different autophagic processes. These findings indicate that the IMM components also participate in mitophagy.^59^

#### 4.1.5 Ambra1

Ambra1, a Beclin 1 interactor, is another mitophagy receptor. After autophagy induction, Ambra1 can gradually transfer from the cytoskeleton to ER and regulate autophagosome nucleation.^60^ The LIR region in Ambra1 can directly bind to LC3 during mitophagy induction. Targeting Ambra1 on mitochondria can promote mitophagy in either Parkin‐dependent or Parkin‐independent pathways. In *Parkin*‐deficient cells, Ambra1 is subject to ubiquitination when Ambra1 is targeted to mitochondria.^61^, ^62^ Ambra1 also promotes ubiquitination of ULK1 through the E3 ligase tumor necrosis factor receptor‐associated factor 6 (TRAF6), indicating that Ambra1 is probably the adaptor for E3 ligases.^63^

It is demonstrated that Parkin can interact with Ambra1, and prolonged mitochondrial depolarization will further enhance their interaction. Moreover, Ambra1 can be recruited to depolarized mitochondria and eventually promote mitophagy, and Parkin translocation can trigger mitophagy through the activation of Ambra1 and the ubiquitination of OMM proteins.^64^

### 4.2 Ubiquitin‐mediated interaction of LC3 adapters

#### 4.2.1 PINK1/Parkin‐mediated mitophagy

PINK1 and Parkin mutations are the most common pathogenic factors of recessive familial PD. PINK1 is a mitochondria‐targeted serine/threonine kinase, while Parkin is a cytoplasmic E3 ubiquitin ligase. PINK1 and Parkin function in the same pathway and to mediate mitophagy in metazoans, with PINK1 acting upstream of Parkin.^65^, ^66^, ^67^, ^68^, ^69^ PINK1 is also regarded as a mitochondrial stress sensor whose function depends on the mitochondrial membrane potential.^70^, ^71^, ^72^

The PINK1‐Parkin pathway may be responsible for regulating the heterogeneity between the healthy and damaged mitochondria and thus mitochondrial homeostasis in cells. Parkin and PINK1 can promote mitochondrial health through several mitochondrial quality control mechanisms, including the turnover of OMM proteins by the proteasome, the generation of mitochondrial‐derived vesicles, and organellar degradation through mitophagy. Under healthy mitochondrial condition, PINK1 is maintained at a low level through complex processing and degradation. But PINK1 is stabilized on OMM with decreased mitochondrial membrane potential, where PINK1 can recruit Parkin to damaged mitochondria.^73^ Thereafter, PINK1 can phosphorylate Parkin at Ser65 and stimulate Parkin's E3 ligase activity^74^; PINK1 also phosphorylates ubiquitin at Ser65, which would then activate Parkin upon binding.^75^, ^76^, ^77^ It is thought that the ubiquitination of OMM proteins by Parkin can initiate the mitophagy process. Parkin can stimulate the attachment of the ubiquitin chain to its substrates through K48 and K63 linkages. Normally, protein degradation can be initiated by K48‐linked ubiquitination, which can initiate passive mitochondrial degradation. The autophagy‐associated adaptors LC3/GABARAP are generally recruited by K63‐linked ubiquitination, which is involved in mitophagy.^78^

After being recruited to the mitochondria, Parkin directs the ubiquitination of various OMM proteins, which can mediate mitochondrial sequestration through interaction with the adaptor proteins on the separation membrane. Several substrates of Parkin have been identified, including mitofusin (Mfn), TOM70, Miro, and Drp1, suggesting that Parkin plays multifaceted roles in the dynamics and biogenesis of mitochondria. p62 is suggested to be recruited to mitochondria along with Parkin; however, p62 is reported to be unnecessary for removing the damaged mitochondria, challenging the role of p62 in the PINK1/Parkin‐mediated mitophagy.^79^, ^80^, ^81^

The mitochondrial fusion GTPase Mfn2 is also a Parkin substrate. Mfn2 can interact with the Miro‐Milton complex on mitochondria and is therefore also related to the transport of mitochondria.^82^ Mfn2 can be phosphorylated by PINK1 and subsequently ubiquitinated by Parkin. As a Parkin receptor, Mfn2 can induce the migration of Parkin to the damaged mitochondria, and the PINK1/Parkin‐mediated Mfn2 ubiquitination may induce mitophagy.^83^, ^84^ However, the ongoing mitophagy in Mfn (−/−) cells suggests that Mfn is not absolutely required for Parkin‐mediated mitophagy.^85^

#### 4.2.2 LC3 adapters: p62, NBR1, OPTN, NDP52, and TAX1BP1

The autophagy adaptor proteins, including SQSTM1 (also called p62), NBR1, optineurin (OPTN), NDP52 (also called CALCOCO2), and TAX1BP1, possess the ubiquitin‐binding domains, which can interact with the ubiquitinated mitochondrial proteins, and the LIR motifs, which can recruit a separation membrane through interacting with LC3, in the selective autophagic degradation of mitochondria.^86^, ^87^, ^88^ Among them, OPTN is one of the most studied adapters for the recruitment of phagophore to the mitochondria mediated by the PINK1/Parkin pathway.^89^ OPTN can be recruited to damaged mitochondria through binding with ubiquitinated OMM proteins, thereby inducing mitochondrial isolation by autophagosome through its interaction with LC3.^90^ TBK1 is activated after recruitment by OPTN, which can then promote mitophagy.^91^, ^92^ Though TBK1 can phosphorylate various adapters, only OPTN and NDP52 have been regarded as its primary substrates.^93^, ^94^ PINK1 has also been shown to play a role in the mitophagy pathway in a Parkin‐independent manner. PINK1 recruits OPTN and NDP52 onto the mitochondria, as well as the subsequent recruitment of autophagy initiation factors, including ULK1 and double FYVE‐containing protein 1 (DFCP1). Interestingly, overexpression of synphilin‐1, an alpha synuclein‐interacting protein, can induce PINK1 accumulation. Later, the PINK1‐synphilin‐1 complex can recruit an E3 ubiquitin ligase (SIAH) to enhance mitochondrial ubiquitination, indicating that E3 ligase other than Parkin can target mitochondria for degradation in distinct ways.^95^

## 5 MITOPHAGY AND NEURODEGENERATIVE DISEASES

Mitophagy often takes place under baseline conditions; however, it can also be promoted under specific physiological conditions. For example, NIX can remove mitochondria from the mature erythrocytes during development, and Parkin and the mitochondrial E3 ubiquitin protein ligase 1 (MUL1) are necessary for the degradation of paternal mitochondria after fertilization in mice.^96^ The differentiation state of stem cells can be affected by the PINK1‐dependent mitophagy.^97^ On the one hand, mitophagy contributes to metabolic changes during the differentiation and the transition from beige to white adipocytes.^98^ On the other hand, mitophagy also exerts great roles in activating the NOD‐like receptor protein 3 (NLRP3) inflammasome, and the FUNDC1‐dependent mitophagy can downregulate platelet activation following acute ischemia/reperfusion injury.^99^, ^100^ As reviewed below, defective mitophagy is frequently observed in neurodegenerative diseases (Table 1).

**Table 1:** Major neurodegenerative disease‐associated proteins which play roles in mitophagy and their mechanisms

Diseases	Proteins that regulate mitophagy	Mechanisms
PD	PINK1/Parkin	1. Ubiquitinate the OMM proteins, which can thereby be recognized by the autophagy receptor proteins, such as OPTN, p62, and NDP52, and induce mitophagy; 2. Regulate mitochondrial fusion and fission to maintain the homeostasis of mitochondria; 3. Induce Mfn ubiquitination to maintain the MMP
	α‐Syn	1. A constituent of the Lewy bodies; 2. Upregulate oxidative stress and impair the mitochondrial function; 3. Activate mPTP through interacting with the VDAC or ANT, disrupt the mitochondrial membrane potential, and initiate mitophagy
	DJ‐1	1. Remove the PD‐associated aggregated p62; 2. Modulate the elimination of endogenous ROS; 3. Function in parallel with the PINK1/Parkin pathway; 4. Regulate the dimerization of α‐Syn
	LRRK2	1. Interact with the regulators of mitochondrial fission and fusion (such as Drp1, OPA1, and Mfn); 2. Regulate the translocation of the CMA complex; 3. Induce the fission of mitochondria through DLP1, then trigger the ULK1‐dependent mitophagy; 4. Initiate the JNK signal by interacting with the JIP3 and MKK4/7; 5. Phosphorylate Bcl‐2, regulate the mitochondrial depolarization and autophagy; 6. Modulate the mitochondrial calcium uptake
AD	PINK1/Parkin	1. Interact in a process to achieve dynamic equilibrium and regulate mitophagy; 2. Modulate the expression PSEN1, which is the major component of the catalytic center in the APP key enzyme γ‐secretase
	Tau	1. Affect the intracytoplasmic translocation of Parkin to the mitochondrial membrane; 2. Regulate Parkin and UCHL1 through the ANT‐1‐dependent ADP/ATP exchange
	Sirtuins	1. SIRT1 can protect neurons from the Aβ aggregation‐induced toxicity; 2. Excessive activation of SIRT2 impairs mitochondrial and microtubule function, leading to mitophagy and Aβ accumulation
HD	PINK1/Parkin	Maintain mitochondrial morphology, ATP levels, and neuronal health, together with the Mfn/MARF, and VDAC/Porin
	GAPDH	Induce the amplified polyglutamine's action, causing mitochondrial dysfunction
	TG2	Cross‐link with Htt, resulting in mitochondrial membrane potential loss, and accumulation of abnormal proteins in the brain
	VCP	Combine with LC3 to cause mitophagy, which seems not to be dependent on the PINK1/Parkin pathway
	HAP1	Regulate the neuronal autophagosome transport
ALS	OPTN/TBK1	1. Affect NF‐κB activity and intracellular transport; 2. Maintain Parkin‐dependent mitophagy homeostasis
	SOD1	Modulate reverse transport of autophagosomes in axons in a PINK1/Parkin pathway‐dependent manner
	VCP	Disrupt the mitophagy balance through the PINK1/Parkin pathway

Note

The dysregulation of mitophagy can lead to damaged mitochondria and accumulation of abnormal protein, which are frequently observed in neurodegenerative diseases. This table lists the major mitophagy‐linked factors associated with neurodegenerative diseases and the potential pathogenic mechanisms.

Abbreviations: α‐Syn, α‐synuclein; Aβ, β amyloid protein; AD, Alzheimer's disease; ADP, adenosine diphosphate; ALS, amyotrophic lateral sclerosis; ANT, adenine nucleotide translocator; ANT‐1, adenine nucleotide transporter 1; APP, amyloid precursor protein; ATP, adenosine triphosphate; CMA, chaperone‐mediated autophagy; DJ‐1 (PARK7), Parkinson disease protein 7; DLP1, dynamin‐like protein 1; GAPDH, glyceraldehyde‐3‐phosphate dehydrogenase; HAP1, Htt‐associated protein‐1; HD, Huntington's disease; JIP3, JNK interacting protein‐1; JNK, c‐Jun N‐terminal kinase; LRRK2, leucine‐rich repeat kinase 2; MARF, mitochondrial assembly regulate factors; Mfn, mitofusin; MKK4/7, mitogen‐activated protein kinase kinase 4/7; MMP, mitochondrial membrane potential; mPTP, mitochondrial permeability transition pore; OMM, outer mitochondrial membrane; OPA1, optic atrophy 1; PD, Parkinson's disease; PSEN1, presenilin 1; SIRT1, Sirtuin 1; SIRT2, Sirtuin 2; SOD1, superoxide dismutase 1; TG2, transglutaminase type 2; Trx/Ask1, thioredoxin/apoptosis signal‐regulating kinase 1; UCHL1, ubiquitin carboxyl‐terminal esterase L1; VCP, valosin‐containing protein; VDAC, voltage‐dependent anion channel.

### 5.1 Mitophagy and PD

Parkinson's disease is characterized by the selective loss of dopaminergic neurons in the pars compacta of the substantia nigra and features clinical phenotypes of rigidity, resting tremor, postural instability, and bradykinesia. Mitochondrial dysfunction has been consistently recognized as an important contributor to the pathogenesis of PD. Major features of mitochondrial dysfunction in PD include ROS overproduction, ATP depletion, mitochondrial DNA depletion, and caspase release. Mitochondrial toxins rotenone and MPTP can impair the mitochondrial function through inhibiting the activity of complex I, whereas suppression of complex I could result in abnormal OPA1 oxidation, which would subsequently lead to abnormality in mitochondrial structure and neurodegeneration of dopaminergic neurons.^101^, ^102^, ^103^

F‐box‐containing proteins, sterol regulatory element binding transcription factor 1 (SREBF1), and WD40 domain protein 7 (FBXW7) have been identified as the critical regulators in the PINK/Parkin pathway of mitophagy.^104^ Moreover, SREBF1 is also one of the risk factors for sporadic PD,^105^ and mitophagy thus may represent a common mechanism linking sporadic and familial PD.^106^, ^107^, ^108^ Mutation in of the F‐box domain is reported to be associated with the early‐onset autosomal recessive PD. FBXW7 is involved in maintaining mitochondria and inducing mitophagy through direct interaction with Parkin, further confirming the significance of mitophagy in the pathogenesis of PD.^109^

Several other genetic mutations, including PINK1, Parkin, DJ‐1, LRRK2, and α‐Syn, have been linked to familial PD. Mutations in LRRK2 and a‐Syn can cause autosomal dominant PD by the gain‐of‐function and possibly proteotoxic mechanisms, while mutations in PINK1 and Parkin can also induce PD through loss‐of‐function mechanism. These genes are associated with mitochondrial function, and the corresponding gene products are also involved in mitophagy. Therefore, these proteins may provide mechanistic links between PD with mitophagy.

#### 5.1.1 PINK1 and Parkin

PINK1 and Parkin are the most extensively studied PD‐associated proteins that are involved in mitochondrial function. PINK1 is encoded by the *PARK6* gene and acts as a serine/threonine kinase targeted to mitochondria,^110^, ^111^ whereas Parkin is encoded by the *PARK2* gene and is an E3 ubiquitin ligase.^112^ Loss of Parkin or PINK1 function results in autosomal recessive PD,^113^ and the PINK1‐dependent initiation of Parkin recruitment to defective mitochondria is regarded as an important regulatory mechanism in mitophagy. Parkin is normally a cytosolic protein, which is recruited to dysfunctional mitochondria in a PINK1‐dependent manner.^114^ Parkin will broadly ubiquitinate and degrade the OMM proteins through the ubiquitin‐proteasome system.^115^

On dysfunctional mitochondria, ubiquitinated OMM proteins produced by Parkin can be recognized by the autophagy receptors, such as OPTN, p62, and NDP52, which can initiate the autophagy process. PINK1 appears to play an incontrovertible role in mitophagy, but Parkin seems to amplify and promote PINK1 effect but itself is not obligatory, since the recruitment of autophagy receptor would take place and mitophagy would be initiated in the absence of Parkin activation.^85^, ^116^ The Parkin‐independent events downstream of PINK1 during the mitophagy process remain largely unknown. For instance, the critical substrates and the E3 ligases promoting the mitophagy‐inducing ubiquitination signals remain to be fully elucidated.

PINK1 and Parkin are essential for maintaining the homeostasis of mitochondria, which is achieved by regulating mitochondrial fusion and fission^117^; at least in part through Mfn ubiquitination. Loss of PINK1 or Parkin would result in the accumulation of Mfn on OMM and impair the mitophagy process. In contrast, loss of Mfn would result in decreased mitochondrial membrane potential, and Parkin recruitment would be blocked by inhibiting the translocase of the OMM import system (TOM complex).^118^ In addition, upregulation of the TOM complex can rescue the mitophagy defect caused by Parkin mutations, suggesting that TOM functions as a significant mediator in PINK1/Parkin‐directed mitophagy.

Our recent studies revealed that on damaged mitochondria, the recruitment of cotranslational quality control factors Pelo, ABCE1, and NOT4 to stalled ribosomes results in NOT4‐mediated polyubiquitination of ABCE1 and that polyubiquitinated ABCE1 (poly‐Ub‐ABCE1) provides a molecular signal for recruiting autophagy receptors to initiate mitophagy.^119^ We provided evidence supporting that the PINK1 pathway is deployed to stimulate translation of nuclear‐encoded respiratory chain (nRCC) mRNAs on mildly damaged mitochondria to promote RC biogenesis and thus mitochondrial repair, revealing a new physiological role of PINK1/Parkin in mitochondrial regulation. However, for severely damaged mitochondria that are beyond repair, the PINK1 pathway is used to direct their clearance by mitophagy. Our results also showed that many of the effects of PINK1 in activating mitophagy, from recruiting C‐I30 mRNP to OMM, cotranslational quality control factors and autophagy receptors to OMM‐associated mRNPs, to the subsequent activation of mitophagy, could occur in the absence of Parkin. This contrasts the prevailing view that the activation of Parkin and subsequent ubiquitination of a battery of Parkin substrates on MOM is essential for PINK1‐activated mitophagy.^120^ Consistent with previous studies, our results indicated that though not required, the presence of Parkin provides an amplifying mechanism to promote PINK1‐directed mitophagy, as both the recruitment of autophagy receptors and the clearance of damaged mitochondria are accelerated in the presence of Parkin. These studies thus identified some of the earliest molecular signals in recruiting the autophagy machinery to damaged mitochondria and revealed a mechanistic connection between ribosome‐associated cotranslational quality control on mitochondrial outer membrane and mitophagy. The identification of altered expression of certain cotranslational quality control factors such as ABCE1 in PD patient brain tissues offered further support for the clinical relevance of these findings.

#### 5.1.2 α‐Syn

α‐Syn, a natively unfolded protein, is located at the presynaptic terminal of CNS and participates in the vesicular release; however, it can be detected in some abnormally conformational structures, such as amyloid fibril, oligomer, and protofibril. α‐Syn is identified as a key constituent of the Lewy bodies, the cytoplasmic inclusions that signify the pathological features of PD.^121^, ^122^ Two autosomal dominant α**‐**Syn gene mutations (namely A53T and A30P) have been associated with PD,^123^ In addition to point mutations, some post‐translational modifications can also induce the toxic phenotype of protein, including ubiquitination, phosphorylation, oxidation, nitration, and the dopamine‐dependent adduct formation.^124^

Studies on the role of α‐Syn in PD have revealed a correlation between aberrant α‐Syn expression and mitochondrial dysfunction. α‐Syn accumulation can elevate oxidative stress and impair the mitochondrial function.^125^ Furthermore, overexpressing α‐Syn in neurons can cause mitochondrial breakup both in vitro and in vivo, which will eventually lead to cell death.^126^ α‐Syn accumulation can open the mitochondrial permeability transition pore (mPTP), disrupt the mitochondrial membrane potential, and subsequently initiate mitophagy. In addition, α‐Syn can activate mPTP through interacting with the voltage‐dependent anion channel (VDAC) or adenine nucleotide translocator (ANT).^127^ A53T mutant of α‐Syn can reside in the mitochondrial membrane and impair mitochondrial function as the monomers and oligomers.^128^ Importantly, α‐Syn can be removed through mitochondrial fission and the activity of Parkin,^129^, ^130^ and its role in activating the autophagy of polarized mitochondria demonstrates that there exists certain link between Parkin and α‐Syn during mitophagy in PD pathogenesis.

#### 5.1.3 DJ‐1

DJ‐1, encoded by the *PARK7* gene, is associated with autosomal recessive PD.^131^, ^132^ DJ‐1 has been regarded as a redox sensor, with potential roles in mitochondrial homeostasis.^133^ The upregulation of DJ‐1 can affect macroautophagy in a cell type‐dependent manner. For instance, the neuroprotection of DJ‐1 in dopaminergic neurons can be suppressed by inhibitors of autophagy and inhibitors of the ERK pathway. DJ‐1 can remove PD‐associated aggregated p62, while DJ‐1 deletion will impede basal autophagy and obstruct the mitochondrial dynamics in mouse embryonic fibroblasts.^134^ DJ‐1 is localized to mitochondria as a constituent of the thioredoxin/apoptosis signal‐regulating kinase 1 (Trx/Ask1) complex, which can modulate the elimination of endogenous ROS.^135^, ^136^ DJ‐1 deficiency will result in H_2_O_2_ accumulation in brain mitochondria, elevated oxidative stress level, and death of DA neurons.^137^

In both fibroblasts and neurons, mitochondrial DJ‐1 level is elevated following oxidative damage in a Parkin‐dependent manner. DJ‐1 overexpression can rescue the phenotype of PINK1‐knockout but not Parkin‐knockout *Drosophila* models, indicating that DJ‐1 may function in between PINK1 and Parkin in the same pathway. To further understand the common features of PINK1, Parkin, and DJ‐1, bioinformatics examinations have been carried out, which indicate that Miro interacts with PINK1 and Parkin, whereas HSPA4 interacts with PINK1, Parkin, and DJ‐1**.** In addition, there are dozens of common interacting factors among PINK1, Parkin, and DJ‐1, most of which are associated with transcriptional regulation. Noteworthily, compared with healthy controls, the expression of these proteins and their associated factors are downregulated in PD patients.^138^ DJ‐1 can also directly interact with α‐Syn, and DJ‐1 mutation associated with PD can induce the accumulation of misfolded α‐Syn in dopaminergic neurons, whereas the upregulation of DJ‐1 can decrease the dimerization of α‐Syn.^139^

#### 5.1.4 LRRK2

Mutations of the leucine‐rich repeat kinase 2 (*LRRK2*) gene are associated with familial and sporadic PD and represent the most significant genetic risk factors for PD. LRRK2‐dependent neurodegeneration processes may involve vesicular trafficking, cytoskeletal dynamics, autophagy, mitochondria dynamics, and calcium homeostasis. Numerous LRRK2 mutations have been investigated in PD, among them, the G2019S mutation marks the most frequent cause of autosomal dominant familial PD,^140^, ^141^ which can also be found in approximately 2% sporadic PD cases. Therefore, elucidating the pathogenicity of LRRK2 may shed light on the molecular mechanisms of PD in general.

Expression of mutant LRRK2 negatively affects mitochondrial health. Endogenous LRRK2 can interact with the regulators of mitochondrial fission and fusion (such as Drp1, OPA1, and Mfn).^142^ In PD patient fibroblasts with LRRK2 G2019S mutation, there is increased susceptibility to MPP^+^‐induced cellular death.^143^ The loss of LRRK2 will disrupt the autophagy pathway and promote the production of autophagosomes.^144^, ^145^ Although CMA may mediate the degradation of LRRK2, but the high levels of wild‐type LRRK2 will obstruct the translocation of the CMA complex, leading to the deficiency of CMA.^146^, ^147^

The expression of LRRK2 G2019S can stimulate mitophagy, and the interaction of LRRK2 with ULK1 may play a vital role in this process. Expression of LRRK2 G2019S can also lead to fission of mitochondria through dynamin‐like protein 1 (DLP1), followed by mitochondrial autophagy by the ULK1‐dependent process. Apart from ULK1, LRRK2 can also interact with the endogenous JIP3 and MKK4/7, working together to initiate JNK signaling. The JNK signaling pathway is involved in the pathogenic mechanism of mutated LRRK2, as demonstrated by the suppression of LRRK2 G2019S‐mediated mitochondrial deficiency by JNK inhibitors.^148^ Furthermore, LRRK2 G2019S can also phosphorylate Bcl‐2 at Thr 56, and the Bcl‐2 mutant will eliminate the mitochondrial depolarization and autophagy induced by LRRK2 G2019s. Therefore, Bcl‐2 may function as a bridge linking the LRRK2 G2019S‐mediated dysregulation of autophagy with mitochondrial disorders.^149^

Parkinson's disease‐related LRRK2 mutations can also activate mitochondrial calcium uptake in cortical neurons and fibroblasts of familial PD patients, presumably through upregulating the expression of mitochondrial calcium uniporter (MCU). Thus, suppressing the ERK1/2‐dependent upregulation of MCU can protect from mutant LRRK2‐induced dendrite shortening and inhibit MCU‐mediated calcium import; consistently, enhancing calcium export from mitochondria is also protective. Therefore, LRRK2‐mediated neurodegeneration includes enhanced susceptibility to mitochondrial calcium overload.^150^ The toxicity of α‐Syn may also be mediated through its interaction with LRRK2 kinase. Common protein interactors may regulate or mediate α‐Syn and LRRK2 interaction in PD.^151^

### 5.2 Mitophagy and AD

Alzheimer's disease is a progressively developing neurodegenerative disease, which is associated with the main clinical symptoms of memory impairment, aphasia, indiscriminateness, cognitive impairment, visual‐spatial impairment, and changes in behavior and personality. Its pathogenic mechanism remains unclear. Extracellular amyloid accumulation will form the senile plaques, and such cellular changes, together with the intracellular hyperphosphorylation of microtubule‐associated tau protein, account for the pathological hallmarks of AD.^152^

Recent studies have found that the development of AD is closely correlated with mitochondrial autophagy defects.^153^, ^154^ In a transgenic mouse model of AD, the amyloid‐β protein is accumulated, accompanied by a cascade of upregulated mRNA levels of mitochondrial autophagy‐associated proteins, such as p62, PARK2, DNM1L, BECN1, BNIP3, PINK1, and LC3. In humans, mice, and even nematodes, Aβ can induce UPR^mt ^and mitophagy in a conserved manner, but the specific mechanism remains to be fully defined. The increases in UPR^mt^ and mitophagy are beneficial, which have been shown to contribute to delaying disease progression and boosting the survival in nematodes.^155^ Moreover, Disrupted‐in‐Schizophrenia‐1 (DISC1) has been identified to act as a newly discovered mitophagy receptor. The downregulation of DISC1, as well as Aβ accumulation in AD, will be mutually reinforced, thus creating a vicious circle. Additionally, DISC1 has been shown to protect synapses from Aβ accumulation‐associated toxicity through promoting mitophagy.^156^

#### 5.2.1 PINK1/Parkin

The PINK1/Parkin pathway is a hotspot in research concerning the effect of mitophagy on AD pathology.^157^, ^158^ In neurons expressing the human amyloid precursor protein (hAPP), the AD‐related mitochondrial stress is found to markedly promote Parkin‐dependent mitophagy. Under normal conditions, more Parkin would be recruited to mitochondrial membrane in AD neurons than in healthy neurons, which has been confirmed in AD patient brain samples. This phenotype can be manifested by the enhanced Parkin‐mediated mitophagy and decreased intracellular Parkin concentration during disease progression. In patient fibroblast models, upregulating Parkin expression and enhancing autophagy can restore mitochondrial membrane potential, reduce PINK1 expression, and hinder mitochondrial accumulation. It is also found that in AD, Parkin could reversely regulate PINK1 through a special way, and the two proteins could interact to achieve dynamic equilibrium. Specifically, Parkin can upregulate the mRNA and protein expression levels of PSEN1, which is one of the major components of the catalytic center in the APP key enzyme γ‐secretase. The APP intracellular domain (AICD) is produced by APP through γ‐secretase‐mediated cleavage, and it can act on FOXO3, eventually upregulating PINK1 level and downregulating the SQSTM/TIMM/TOMM signaling.^159^

#### 5.2.2 Tau

In the case of human wild‐type full‐length Tau (hTau), its intracellular accumulation will affect the mitochondrial membrane potential, thereby inducing mitochondrial maladjustment and dysfunction. Besides, it was recently found that hTau can prevent the intracytoplasmic translocation of Parkin to the mitochondrial membrane and inhibit mitophagy in nematode models, while such effect is independent of the changes in membrane potential. On the other hand, the NH2‐terminal fragment of tau can regulate Parkin and UCHL‐1 through inhibiting the ANT‐1‐dependent ADP/ATP exchange, suppress mitochondrial autophagy, and mediate synaptic degeneration in AD.^160^

#### 5.2.3 Sirtuins

There are also other genes and proteins that can act as a bridge between mitophagy and the progression of AD. The sirtuin family is comprised of seven sirtuins. Three of them have been confirmed to be the mitochondrial sirtuins that are closely related to the mitochondrial performance.^161^ Resveratrol has been indicated to upregulate SIRT1 expression, thereby protecting neurons from the Aβ aggregation‐induced toxicity. It is shown that the antiinflammatory and antioxidant properties of resveratrol can delay the development of AD, and resveratrol can remarkably enhance mitochondrial autophagy.^162^ Resveratrol can also markedly reverse Aβ effects, manifested by elevating the levels of LC3‐II/LC3‐I, Parkin, and Beclin 1. Additionally, it can reduce the positioning of LC3 and TOMM20, and in the meantime, it modulates BNIP3‐ and NIX‐related processes.^163^, ^164^, ^165^ In AD, excessive activation of the tubulin deacetylase SIRT2 will result in mitochondrial dysfunction and the ensuing mitophagy. On the other hand, pharmacological inhibition or gene knockdown of SIRT2 will improve microtubule function, restore mitochondrial homeostasis, improve autophagy, and reduce Aβ accumulation.

In general, accumulating evidences have verified the presence of mitochondrial dysfunction and abnormal mitochondrial autophagy in the brains of AD patients, which are closely related to disease occurrence and progression. By regulating mitophagy and improving mitochondrial function, it may help delay the pathological process, which is of great significance for the eventual establishment of clinical treatment strategies.

### 5.3 Mitophagy and HD

HD is an autosomal dominant neurodegenerative disease, which is frequently seen in middle‐aged people. Its clinical symptoms primarily feature dance‐like movements, followed by cognitive impairment, and even loss of behavioral and language ability. The progression of HD follows an intractable course, and the survival time for patients is generally 10‐20 years. The main cause of HD is the mutation in the huntingtin (Htt) gene located on chromosome 4. Specifically, the CAG repeat in exon 1 of Htt is expanded, resulting in expansion of N‐terminal polyglutamine of the mutant Htt protein. Mutant Htt protein gradually accumulates in cells to form the macromolecular aggregation, which will induce cytotoxicity. Mutant Htt can interfere with the normal function of nerve cells through a number of mechanisms, including mitochondrial dysfunction, oxidative stress, amino acid metabolism, protein transport, and the apoptosis signaling. The presence of mHtt can result in neurological abnormalities or regression.^166^, ^167^, ^168^

Increasing evidence suggests that mutant Htt can damage mitochondria, leading to energy metabolism disorders, oxidative stress, and release of proapoptotic factors.^18^, ^169^ The aberrant morphology of mitochondria can be observed in the *Drosophila* model of HD. In addition, mutant Htt is found to cause formation of spherical mitochondria in a nonapoptotic state, and it can also repress mitophagy, resulting in the impaired mitochondrial clearance.^170^

#### 5.3.1 PINK1/Parkin

In the *Drosophila* model of HD, increased PINK1 expression can suppress the mitochondrial morphological abnormalities and accumulation, while overexpression of PINK1 reduces neuronal vacuolization, restores ATP levels, and reduces mortality in adult *Drosophila*. These results suggest that PINK1 can protect neurons against mutant Htt toxicity. However, the effect of PINK1 is dependent on Parkin activity, which requires the involvement of the Mfn/mitochondrial assembly regulate factors (MARF) and VDAC/Porin. Similar results can be obtained in the mouse model of HD, in which mutant Htt can reduce the targeting of mitochondria to autophagosomes, which can be reversed by the overexpression of PINK1.^18^

#### 5.3.2 GAPDH

Polyglutamine expansion is an important pathogenic mechanism of HD. Recent studies revealed that the amplified polyglutamine can cause mitochondrial dysfunction, which is mainly characterized by morphological abnormalities, blockade of respiratory function, reduced ATP production, and the release of proapoptotic factors. Such mitochondrial damage is at least partly ascribed to the inactivation of glyceraldehyde‐3‐phosphate dehydrogenase (GAPDH) on mitochondria. This is followed by the inhibition of GAPDH‐mediated mitophagy and the accumulation of impaired mitochondria in cells. GAPDH selectively binds to mitochondria, and overexpression of GAPDH can restore mitochondrial function.^171^

#### 5.3.3 Transglutaminase type 2

Transglutaminase type 2 (TG2) is closely related to mitochondrial clearance and homeostasis. Its expression in the brain of HD patients is elevated, and it cross‐links with Htt, resulting in mitochondrial membrane potential loss, accumulation of abnormal proteins, and neuronal apoptosis in the brain. On the other hand, pharmacological inhibition or gene knockdown of TG2 can protect against neurodegeneration in HD, and TG2 may be one of the causes of progressive death of HD neurons.^172^

#### 5.3.4 Valosin‐containing protein

Recent studies have shown that in the HD model, valosin‐containing protein (VCP) is localized on mitochondria where it specifically binds mutant Htt,^173^ while VCP accumulation can lead to excessive activation of mitophagy, thus inducing neuronal damage. This effect is caused by the interaction of its LIR with LC3, and it seems not to be dependent on the PINK1/Parkin pathway, but the detailed mechanism remains unclear. A peptide HV‐3 can block VCP translocation to mitochondria and inhibit excessive mitophagy damage. It has been shown to exhibit the neuron protective effect in the HD model. Therefore, inhibiting the interaction of VCP with mutant Htt may be a new strategy for treating HD.^173^

Impaired axonal transport of autophagosomes has also been suggested to be responsible for the inability of abnormal mitochondria to be cleared in HD. The Htt‐associated protein‐1 (HAP1) and Htt are shown to regulate neuronal autophagosome transport.^170^ Depletion of Htt increases the autophagosomes containing mitochondrial fragments, indicating that mitochondrial clearance is reduced. However, the authors found that Htt would not alter the flux of mitophagy. Inhibiting the α‐tubulin deacetylase HDAC6 is also considered a potential avenue for treating HD, but the mechanism remains unclear, and studies suggest that HDAC6 may block mitophagy.^174^ Various studies have revealed reduced mitophagy in the brain of HD patients, which lead to subsequent dysfunction.^171^ Currently, developing treatment strategies for HD by rebuilding stable mitophagy balance has attracted great interests.

### 5.4 Mitophagy and ALS

ALS is a neurodegenerative disease that develops in adulthood, accompanying the loss of motor neuron disease (MND) and upper motor neuron (UMN). The pathological hallmark of ALS is progressive motor dysfunction, but the sensory function is found to be unaffected. Nonetheless, it has been found in recent years that the neurodegeneration in ALS is not limited to the motor system; instead, it has involved the sensor, linguistic, behavioral, and other cognitive domains. Some patients may have mild cognitive impairment and even significant frontotemporal dementia.

The pathogenesis of ALS has been extensively explored. More than 30 gene mutations have been reported to be associated with ALS so far, and SOD1, OPTN, TBK1, VCP, and C9ORF72 have become the research hotspots. In ALS models, the accumulation of damaged mitochondria can be detected by live cell imaging. Mitochondrial homeostasis can be reconstituted by inhibiting OPTN or TBK1 mutations, as well as pharmacological inhibition or genetic knockdown of PINK1 or Parkin. Altering TBK1/OPTN can significantly improve neuronal function and block disease progression. These data support the potential role of mitochondrial autophagy in ALS.^89^, ^91^, ^175^

#### 5.4.1 OPTN/TBK1

Three ubiquitin‐LC3‐binding autophagy receptors involved in mitophagy have been studied, including OPTN, CALCOCO2, and TAX1BP1. OPTN mutations are well known to underlie the pathogenesis of glaucoma and ALS.^176^ It was found that the time course of recruitment of these receptors to the mitochondria is similar, but only OPTN can trigger the subsequent formation of autophagosomes around damaged mitochondria. Moreover, OPTN mutations have been shown in previous studies to enhance NF‐κB activity and impede intracellular transport. Besides, mutations are suggested to activate the inflammatory factors, which also contribute to disease progression.^177^

As found in recent studies, OPTN will translocate to damaged mitochondria, which is dependent on Parkin ubiquitination of mitochondria.^175^ Time course studies during mitophagy reveal that the recruitment of Parkin, that of OPTN, as well as the formation of autophagosomes occur at 30, 45, and 60 min after injury, respectively. Besides, OPTN can bind with mitochondria transiently in the absence of Parkin. But such binding would be more stable in the presence of Parkin. Subsequently, DFCP1 can translocate to mitochondria and recruit LC, and the LIR of OPTN will mediate the formation of autophagosomes around the scathing mitochondria, indicating that OPTN is important in the Parkin‐mediated mitophagy. However, this process will be hindered, and mitochondrial accumulation will be impaired if the endogenous OPTN is depleted. Notably, the wild‐type mice with OPTN siRNA resistance can withstand such damage, but mice with the ALS‐related OPTN mutation (E478G) cannot. The ALS‐related OPTN UBAN mutant has been utilized to block the translocation of OPTN to the mitochondria, which will lead to abnormal mitophagy. The result thus links OPTN with ALS through the mitochondrial degradation efficiency, demonstrating the significance of mitophagy to ALS.

The effect of OPTN translocation to mitochondria to induce mitophagy is consistent with the recruitment of its upstream kinase TBK1. Specifically, the OPTN‐mediated mitophagy depends on the phosphorylation of its serine 177 by TBK1. Similar to OPTN, TBK1 mutations can also cause ALS. Pharmacological inhibition of TBK1 or expression of ALS‐associated TBK1 mutants can reduce the mitophagic efficiency of damaged mitochondria. In conclusion, both OPTN and its upstream protein TBK1 are required for normal mitophagy homeostasis.^178^ As shown in these studies, TBK1 phosphorylates OPTN, enhancing its ability to bind to ubiquitinated mitochondria, suggesting that TBK1 may also be one of the potential targets for treating ALS.^94^

Furthermore, evidence suggests that the TBK1/OPTN axis is linked to the PINK1/Parkin pathway, which can mediate autophagosome production and the clearance of impaired mitochondria. Both inhibition of OPTN or TBK1 and knockdown of PINK1 or Parkin can ameliorate mitophagy in ALS. However, the knockout of PINK1 or Parkin would exert no significant neuroprotection. Reasons may be that PINK1/Parkin mediates the production of autophagosomes and can be seen anywhere in the neuron, but the engulfment of mitochondria by autophagosomes is dependent on the TBK1/OPTN pathway.^179^

#### 5.4.2 SOD1

SOD1 is one of the most extensively investigated ALS‐related genes. Its mutations cause accumulation of hydroxyl radicals, trigger cytotoxicity, and contribute to ALS. Notably, it has recently been reported that SOD1 may indirectly participate in regulating the mitochondrial homeostasis in ALS.^180^, ^181^

On the other hand, it is found that in the SOD1 model of ALS, the flux of mitophagy is decreased. Mitochondria quality control (MQC) can restore mitophagy and reduce the accumulation of impaired mitochondria, which may be ascribed to the potential effect of SOD1 in blocking the reverse transport of autophagosomes in axons. It is suggested that SOD1 relies on Parkin to reduce the rate‐limiting factor mitochondrial Rho‐GTPase 1 (Miro1), whereas overexpression of Miro1 and inhibition of PINK1 can reverse the autophagosome trafficking defect resulted from the SOD1 mutations. It is suggested that mutant SOD1 would impair axonal transport in a PINK1/Parkin pathway‐dependent manner.^182^ In the G93A mutant SOD 1 transgenic (SOD1‐G93A) mouse model, mitochondria can recruit P62 and activate mitophagy. In that model, the mitochondrial defects are manifested as a variety of protein changes, such as ubiquitin ligase Parkin, mitochondrial biogenesis protein PGC‐1α, and Miro1. Knocking down Parkin can reverse such negative effect and improve the pathological symptoms of ALS. But the effect is not sustained and may be compensated by other ubiquitinating enzymes.^169^, ^183^, ^184^

In addition, VCP also exhibits some properties similar to OPTN; for instance, it relies on the Parkin‐mediated ubiquitination to be recruited to the mitochondria and is involved in mitophagy. The ALS‐related VCP mutations will disrupt the mitophagy balance through the PINK1/Parkin pathway, thereby affecting the clearance of abnormal mitochondria.^185^

At present, the pathogenesis of ALS remains to be fully understood, and the existing hypotheses include oxidative stress, mitochondrial dysfunction, excitotoxicity, and neuroinflammation.^186^, ^187^, ^188^ Notably, research on mitophagy, inflammation, and their interaction has become a hotspot. Improving mitochondrial autophagy and restoring mitochondrial homeostasis may offer potential treatment for ALS.

## 6 CONCLUSIONS

Neurodegenerative diseases are associated with protein turnover, and protein aggregation is involved in the cellular pathology of many neurodegenerative diseases**.** Mitophagy can partially account for the mechanisms of cellular homeostasis; therefore, appropriate mitophagy level is of great significance to reduce the aggregation of abnormal proteins and to stimulate organelle removal. For the sake of protecting neurons, it is necessary to maintain the mitochondrial function and promote the degradation of damaged mitochondria. The regulation of mitophagy is suggested to be one of the therapeutic strategies for some neurodegenerative diseases. The activation of mitophagy has been shown to improve neurodegenerative diseases phenotypes and offer neuroprotection. Several therapeutic tools have been confirmed to increase mitophagy, such as the regulators of PINK1/parkin, metformin, and resveratrol.^189^ In recent experiments, some drugs are also thought to delay the disease progression, which target the sirtuins family. Sirtuin activating compounds (STACS) or NAD precursors such as NR/NMN can increase mitophagy through regulating sirtuins.^189^, ^190^ Many natural compounds with bioactivation are gradually being discovered. Some antibiotics or plant ingredients also induce mitophagy, such as actinomycetes.^191^ It is associated with mitochondrial autophagy in neural stem cells, which may be mediated by ribosome depletion.^192^

Nevertheless, both excessive and reduced mitophagy may be harmful, making mitophagy a double‐edged sword. Mitochondrial autophagy plays an important role in the self‐maintenance of the nervous system, but only the role of PINK1/Parkin pathway in the regulation of neurodegenerative diseases has been well specified so far, and some results are still controversial. The current results only partially reveal the role of mitophagy mediated by the PINK1/Parkin pathway in PD, while there are few studies on AD and HD, and many questions have not been clarified.^193^, ^194^ NIX/BNIP3L and FUNDC1 mainly regulate mitochondrial autophagy under hypoxic conditions, but the regulation of mitochondrial autophagy under hypoxic and ischemic conditions needs to be further explored.^195^

Consequently, simply elevating the mitophagy level is not a feasible approach, and there are still some future challenges; for instance, how to optimize the mitophagy activity for neuron protection to treat neurodegenerative diseases, how to elucidate the common molecular mechanisms regarding the pathogenesis of neurodegenerative diseases, and how to clarify the interactions among mitophagy, mitochondrial metabolism, and mitochondrial dynamics. Researches on the molecular mechanisms related to mitophagy should be strengthened, and technologies that can visually and real‐time monitor mitochondrial morphological changes should be developed. In addition, more specific agents that target mitophagy can be applied to cultured neurons or animal models of neurodegenerative diseases to observe the relationship between mitophagy and diseases.

## CONFLICT OF INTEREST

The authors declare no conflict of interest.
